# Language difficulties are a shared risk factor for both reading disorder and mathematics disorder

**DOI:** 10.1016/j.jecp.2020.105009

**Published:** 2021-02

**Authors:** Margaret J. Snowling, Kristina Moll, Charles Hulme

**Affiliations:** a Department of Experimental Psychology and St. John’s College, University of Oxford, Oxford OX1 3JP, UK; b Department of Child and Adolescent Psychiatry, Psychosomatics, and Psychotherapy, University Hospital, Ludwig-Maximilians University Munich, 80336 Munich, Germany; c Department of Education, University of Oxford, Oxford OX1 3JP, UK

**Keywords:** Reading disorder, Mathematics disorder, Comorbidity, Developmental language disorder

## Abstract

Disorders of reading and mathematics co-occur at greater than chance rates, but they are often studied separately. This article reports the reading and arithmetic outcomes at 9 years of age from a longitudinal study of 224 children at high risk for dyslexia. Using a cutoff at the 10th centile, groups of children with reading disorder (RD), mathematics disorder (MD), and comorbid reading disorder and math disorder (RD&MD) were identified. The risk of these disorders was elevated in children selected in preschool with language difficulties or at family risk for dyslexia. There was a high degree of comorbidity between RD and MD, and many cases—particularly in the comorbid group—also reached the diagnostic threshold for developmental language disorder (DLD). On measures of language, phoneme awareness, rapid automatized naming (RAN) digits, and rated inattention, there was a stepwise pattern: RD > MD > RD&MD. Poor language was associated with each disorder and appears to be a cognitive risk factor for RD, MD, and RD&MD. These findings suggest that there are shared genetic risk factors operating for both RD and MD.

## Highlights

• Disorders of language (DLD), reading (RD) and mathematics (MD) are highly comorbid.
• RD and MD is associated with familial dyslexia and preschool language difficulties.
• The comorbidity between RD and MD arises because of shared risk factors.
• Children with RD should be assessed for MD and vice versa.
• Interventions for MD should take account of likely language problems.

## Introduction

Children with poor language skills at school entry are at high risk for low educational attainment, and those with developmental language disorder (DLD) are very likely to experience difficulties in learning to read (Snowling et al., 2019) and in becoming numerate (Durkin, Mok, & Conti-Ramsden, 2013; see also Bishop, Snowling, Thompson, Greenhalgh, & and CATALISE-2 Consortium, 2017). Problems of reading and mathematics are grouped together as “specific learning disorders” and separately from language disorders in the Diagnostic and Statistical Manual of Mental Disorders (DSM-5; American Psychiatric Association, 2013). More broadly within the DSM-5 classification system, reading, mathematics, and language disorders are categorized as neurodevelopmental disorders; such disorders are highly heritable, have an early onset during childhood, and affect the course of development (Thapar, Cooper, & Rutter, 2017). It follows that it is appropriate to study them developmentally. However, although the frequent co-occurrence of reading problems (reading disorder or dyslexia) and mathematical difficulties (mathematics disorder or dyscalculia) has been highlighted by population surveys (Moll, Kunze, Neuhoff, Bruder, & Schulte-Körne, 2014) and is highly stable over time (Koponen et al., 2018), there are relatively few longitudinal studies of these groups. However, Purpura, Hume, Sims, and Lonigan (2011) argued that early literacy measures are sensitive predictors of numeracy development, and Joyner and Wagner (2019) suggested that including mathematical skills as predictors of reading can improve the accuracy of identification of reading disabilities. A shortcoming of such proposals, however, is that the possible effects of poor language skills on learning to read and to develop number skills are not made explicit.

Against this background, and given the growing interest in the causes of comorbidity (Moll, Snowling, & Hulme, 2020), it is surprising that neither classification systems nor surveys highlight the fact that poor language may be a common risk factor for both reading disorder (RD) and mathematics disorder (MD). More generally, in comparison with the large amount of research linking language and reading disorders (Bishop & Snowling, 2004), there is a dearth of studies investigating numeracy skills in children with DLD and findings are inconclusive (Cross, Joanisse, & Archibald, 2019). Here we report data from a study of children at risk for reading difficulties, because of a familial risk of dyslexia and/or preschool language difficulties, followed from preschool 9 years of age when reading and mathematical skills were assessed. The study offers the opportunity to examine the association between disorders of reading, mathematics, and language among children selected as being at risk for dyslexia in preschool, before they were in receipt of formal instruction in reading and mathematics.

According to Caron and Rutter (1991), the co-occurrence of two disorders can arise because of shared risk factors, because one disorder is the developmental precursor of another, or because one disorder confers risk of a second disorder. In practice, most investigations of the comorbidity between RD and MD have used a cross-sectional design to examine the cognitive deficits associated with each group and the interaction between them (see Swanson & Jerman, 2006, for a review). Although this approach can help to identify shared and specific risk factors associated with two disorders, it is difficult to differentiate different causes of comorbidity in the absence of longitudinal data. The current study examined both familial risk of RD and preschool language difficulties as risk factors for RD and MD, their comorbidity with each other, and their comorbidity with DLD. Using data from the first waves of this study, we previously reported the predictors of reading and arithmetic shortly after school entry: language at 3½ years was an indirect predictor of reading (decoding) at 5½ years (its effects are mediated by phoneme awareness and letter knowledge at 4½ years; Hulme, Nash, Gooch, Lervag, & Snowling, 2015). Arithmetic at 6½ years was also predicted by language at 3½ years, in this case together with executive function (its effects were mediated by counting and number knowledge at 4½ years; Moll, Snowling, Gobel, & Hulme, 2015; see also LeFevre et al., 2010). Although it is well established that reading, mathematics, and language are highly heritable skills (Haworth & Plomin, 2010), studies of children at familial risk for dyslexia do not generally report numeracy outcomes (cf. van Bergen, de Jong, Maassen, & van der Leij, 2014), nor do they include comparison groups of children with DLD. Furthermore, to our knowledge, no study has investigated the overlap of RD and MD with DLD.

Relevant to this issue are studies by Willcutt, Peterson, and colleagues (Peterson et al., 2017, Willcutt et al., 2013) using large sets of data from twins in the Colorado Learning Disabilities Study. Together, these point to the importance of verbal ability (a construct related to language) as a risk factor for RD, MD, and their comorbidity. In dimensional analyses, verbal comprehension, verbal working memory, and symbol processing speed predicted both reading and math; in addition, language-related skills, namely phoneme awareness and rapid automatized naming (RAN), predicted reading (see also Cirino, Fuchs, Elias, Powell, & Schumacher, 2015). There has been less consistency in the measures used to predict mathematical attainment and MD. Willcutt et al. (2013) found set shifting in the Wisconsin Card Sorting Test (a measure of executive function) to be a concurrent predictor of mathematics. In contrast Peterson et al., 2017, Cirino et al., 2015 reported that working memory deficits characterized children with MD. The study by Cirino et al. also measured foundational numerical concepts that, together with measures of processing speed and nonverbal processing, were associated with MD. Finally, in one of the few studies reporting longitudinal data, Koponen et al. (2020) investigated the cognitive predictors (assessed in Grade 1) of the covariance between reading and arithmetic fluency (assessed in Grade 2) in 200 Finnish children. Naming speed (RAN) was the strongest predictor of the shared variance between measures of reading and arithmetic. Consistent with this, Korpipää et al. (2017) showed that from Grade 1 and Grade 7, reading and arithmetic share common processes and that RAN, verbal working memory, nonverbal reasoning, letter knowledge, and counting account for most of the covariation between skills.

In summary, verbal ability is reported as a domain-general factor that predicts reading and mathematics when both are diagnosed using fluency measures, and it appears that RAN (the ability to name familiar items at speed) is a shared risk factor for both RD and MD. In addition, phoneme awareness is a specific predictor of reading, and deficits in visual–spatial memory, perceptual speed, working memory, and executive function (set shifting and interference) are common in children with MD (Haberstroh and Schulte-Körne, 2019, Peng et al., 2018). A smaller number of studies (Göbel and Snowling, 2010, Landerl et al., 2004, Landerl et al., 2009, Raddatz et al., 2017) have included numerosity-specific predictors; these were not included in the current study. Rather, within the multiple-risk framework of Pennington (2006), our broad aim was to test the common liabilities model of the comorbidity between RD and MD, building on the findings of Peterson et al., 2017, Willcutt et al., 2013.

Drawing on data from different waves of our risk study, we report assessments of reading and mathematical skills at 9 years of age that we used to identify children with RD, MD, and comorbid RD and MD (RD&MD), and we examine the association with DLD identified a year earlier at 8 years of age. We proceed to compare the cognitive profiles of the groups using children without disorders from the same sample as a benchmark age-matched comparison group. We predicted an elevated risk of MD, RD, and RD&MD in children at familial risk for dyslexia and in children with preschool language difficulties. We also expected the rate of RD&MD to be particularly high in children with concurrent DLD. In terms of cognitive profiles at 6 years of age, we expected RD and MD to be associated with shared deficits in language skills and naming speed (RAN), whereas children with pure RD were expected to show deficits in phoneme awareness and children with pure MD were expected to show deficits in visual–spatial skills and executive function. We also examined parental ratings of inattention that we expected to be associated with both disorders.

We tested the following specific hypotheses:

1. There will be substantial rates of comorbidity among RD, MD, and DLD.
2. There will be an elevated risk of MD in children at family risk for dyslexia and in children with preschool language difficulties. The risk will be particularly high in children with concurrent DLD.
3. Children with RD will show deficits in phoneme awareness and RAN.
4. Children with MD will show deficits in visual–spatial skills and executive function.
5. Both RD and MD will be associated with shared deficits in verbal skills, defined by a language factor, in verbal processing speed and parental ratings of inattention.

Method

### Participants

The sample was from the Wellcome Language and Reading Project that followed the development of children at high risk for dyslexia from 3½ to 9 years of age. Ethical approval for the study was provided by the University of York, Department of Psychology Ethics Committee, and the NHS Research Ethics Committee. Parents provided informed consent for their children to participate. At the beginning of the study, we recruited a sample of 260 children aged 3½ years who had been volunteered by their families to take part. Children were assessed by trained testers and classified using a two-stage process to determine (a) whether they were at family risk for dyslexia and (b) whether they had a preschool language impairment placing them at risk for DLD (see Nash, Hulme, Gooch, & Snowling, 2013, for details). A total of 71 children with no history of language problems or other risk factors formed a control sample, the typically developing (TD) control. There was a small amount of attrition; data from all children who remained in the sample at 9 years of age (*N* = 224) are included in the current analyses.

### Classification of attainments at 9 years of age

Reading and number skills are continuously distributed in the population, and there is no clear division between “typical” and “impaired” levels of performance (Branum-Martin, Fletcher, & Stuebing, 2013). However, when considering whether an *individual* is functionally impaired, a cutoff criterion needs to be used. Here, we considered children to be impaired if reading and/or number skills fell below the 10th centile of the typical control group on a factor score with high loadings from three domain-specific tests.

To define a reading factor, we used three subtests from the Diagnostic Test of Word Reading Processes (Forum for Research Into Language and Literacy, 2012) comprising 30 regular words, 30 exception words, and 30 nonwords. To define an arithmetic factor, we used three tests: Numerical Operations subtest from the Wechsler Individual Achievement Test (WIAT-II; Wechsler, 2005), One Minute Addition, and One Minute Subtraction (see below for details). We then used the standardized factor scores to define the score in each domain that represented the cutoff at the 10th centile of the TD control sample below which performance was defined as “impaired.” Using these criteria, 25% of the sample (*n* = 56) was defined as reading impaired (RD), and 23% of the sample (*n* = 51) was defined as math impaired (MD). There was also a high degree of comorbidity—among children with RD, 60% also had MD; among children with MD, 65% also had RD.

### Tests and procedures

Each child was administered a large battery of tests during a 2-h session. The tasks are described fully elsewhere (Snowling et al., 2019); here, brief details are provided for the tests used to classify the children into diagnostic groups at 9 years of age and for the cognitive measures used at 6½ years of age, together with parent and teacher ratings of behavior and attention. For each cognitive domain tapped by more than one measure, a factor score was derived for use in analyses.

#### Nonverbal ability

The Block Design and Matrix Reasoning subtests from the Wechsler Intelligence Scale for Children (WISC-IV; Wechsler, 2003) were given (split-half reliability: Block Design = .89, Matrix Reasoning = .89) at 8 years of age.

#### Language (6½ years of age)

For receptive grammar, sentence–picture matching from the Test for Reception of Grammar (TROG-2 ; Bishop, 2003) measured language comprehension (*α* = .88).

For expressive grammar, an experimental sentence imitation test required repetition of 20 sentences: 10 (5 long and 5 short) containing transitive verbs and 10 (5 long and 5 short) containing ditransitive verbs (*α* = .78). The score is the number of sentences repeated correctly.

For morphological inflection, the Word Structure subtest from the Clinical Evaluation of Language Fundamentals (CELF-4; Semel, Wiig, & Secord, 2006) required children to use a given word (or words) to describe a picture. Responses were credited according to guidelines (*α* = .78–.86).

For vocabulary, the Expressive Vocabulary subtest from the CELF-4 measured naming ability (with five extension items added to avoid ceiling effects) (*α* = .66). In addition, the Receptive One Word Picture Vocabulary Test (ROWPVT; Brownell, 2000) measured vocabulary knowledge (*α* = .95).

For listening comprehension, children listened to recordings of two short stories and answered 17 questions about them. Questions required children to make both literal responses and inferences (*α* = .79).

#### Phonological awareness

Children completed phoneme deletion from the York Assessment of Reading for Comprehension (YARC; Hulme et al., 2009*)* at 5½ years of age and with extension items to avoid ceiling effects at 6½ years of age) (*α* = .95).

#### Rapid automatized naming (6½ years of age)

Children named an 8 × 5 array of 40 stimuli as quickly as possible for two trials of RAN digits. The RAN rate was calculated as the mean number of items named per second (the lower bound estimate of reliability was .74; test–retest correlation for RAN digits at 6½ and 8 years of age).

#### Executive function (6½ years of age)

For selective attention, children were assessed using a visual search task, the Apples task (Breckenridge, 2008). A visual search efficiency score [(hits: total targets correctly identified − commission errors)/60 s] was calculated (stability: *r* = .59).

For visual–spatial memory, the Block Recall subtest from the Working Memory Test Battery for Children (WMTB-C; Pickering & Gathercole, 2001) was given (*α* = .63). The examiner tapped a sequence of blocks, and children recalled this by tapping the blocks in the same order. The trials began with short (three-item) sequences and gradually increased until two trials of the same length sequence were incorrect. The number of correct trials was recorded (maximum = 52).

#### Speed of processing (6½ years of age)

For simple reaction time, children’s response time to a picture of a bug was measured with a computer-presented Go/NoGo task in which a ladybird also appeared and was to be ignored. After a fixation cross, the stimulus was presented for 500 ms, after which the screen was blank. Children had 2000 ms from stimulus onset to make their response. Children completed three practice trials, followed by 30 “go” trials.

#### Attention (6½ years of age)

The Strengths and Weaknesses of ADHD Symptoms and Normal Behavior Questionnaire (SWAN; Swanson et al., 2012*)* was completed by parents and teachers*.* Items mapped onto the symptoms of attention-deficit/hyperactivity disorder (ADHD) and included nine items tapping inattention and 9 items tapping hyperactivity/impulsivity. For each item, respondents compare children’s attention/behavior with those of peers using a 7-point scale (maximum score = 126). A low score reflected weaknesses in attention/behavioral skills.

#### Reading outcome (9 years of age)

The Diagnostic Test of Word Reading Processes (DTWRP; Forum for Research Into Language and Literacy, 2012) was given. This comprises sets of regular and irregular words and nonwords to be read aloud. For a composite score, reliability was *α* = .97. The reliabilities for individual tests were as follows: regular words = .88, irregular words = .83, and nonwords = .77.

#### Mathematics outcome (9 years of age)

For numerical skills, the Numerical Operations subtest from the WIAT-II (Wechsler, 2005) was given. The first items measure number knowledge (i.e., identifying numbers, number sequence, and transcoding), followed by written calculation problems involving addition, subtraction, multiplication, and division of increasing difficulty (starting with single-digit calculations and followed by multidigit calculations) (*α* = .88).

For timed arithmetic, children completed as many single-digit additions/subtractions as possible within 1 min (maximum = 30 per subtest). All operands and answers were below 20. Items 1 to 20 included only single digits as operands and answers (e.g., addition: 2 + 5; subtraction: 7–3), and Items 21 to 30 involved crossing the decade (e.g., addition: 5 + 7; subtraction: 14–6). The number of correctly solved items per second (efficiency) was calculated for each subtest (test–retest reliability: addition = .92 and subtraction = .88).

## Results

The means and standard deviations for the four groups (TD control, RD, MD, and RD&MD) on standardized measures are shown in Table 1.

**Table 1:** Means (and standard deviations) of RD, MD, and RD&MD compared with TD outcome at 9 years of age.

	TD outcome (*n* = 150)	RD (*n* = 22)	MD (*n* = 18)	RD&MD (*n* = 33)
Age (months)	111.29 (5.62)	109.59 (5.96)	104.5 (5.64)	105.66 (5.78)
PIQ^a^	107.96 (13.29)_1_	101.77 (12.30)_1,2_	89.17 (10.55)	93.27 (15.73)_2_
Block Design	11.89 (2.53)_1_	11.5 (2.30)_1_	9.28 (2.44)_2_	9.85 (2.67)_2_
Matrix Reasoning	10.76 (2.80)_1_	9.09 (2.78)_1,2_	7.11 (2.14)_2,3_	7.91 (3.02)_3_
Reading^b^	111.24 (10.56)_1_	88.55 (6.91)_2_	105.89 (9.52)_1_	85.36 (11.07)_2_
Number^c^	109.45 (15.28)	96.00 (10.08)_1_	84.06 (13.70)_1,2_	77.94 (10.97)_2_
Reading score^d^^,^^e^	.48 (.33)_1_	−.91 (.51)	.23 (.39)_1_	−1.56 (1.22)
Math score^e^^,^^f^	.49 (.72)	−.21 (.43)	−1.26 (.47)_1_	−1.38 (.50)^1^

*Note.* Values with the same subscript do not differ. TD, typically developing; RD, reading disorder; MD, mathematics disorder; RD&MD, comorbid RD and MD; PIQ, Performance IQ.

a Wechsler Intelligence Scale for Children (WISC-IV) Block Design and Matrix Reasoning subtests, scaled score (at 8 years of age).

b Diagnostic Test of Word Processes, standard score.

c Wechsler Individual Achievement Test (WIAT II) Numerical Operations subtest, standard score.

d Latent factor (regular word, exception word, and nonword reading).

e Factor scores derived relative to outcomes at 9 years of age for TD controls recruited at 3½ years of age.

f Latent factor (addition, subtraction, or written sums).

The group with typical outcomes (TD control) obtained higher scores on all measures than the three clinical groups. In reading, standard scores on the reading measure (DTWRP) indicate that both RD groups were impaired to a similar extent, whereas the MD group was not significantly worse than the TD control group. In arithmetic, standard scores on the Numerical Operations subtest suggest that the RD&MD group was marginally worse than the MD group but not significantly so. Although the RD group performed less well on average than the typical group, the mean of the RD group was in the average range; the group difference between the RD and MD groups, however, was not statistically significant. The comorbid group was worse in reading than in number skills; importantly, this group’s performance was not significantly different from that of the RD group in reading and not significantly different from that of the MD group in arithmetic. There were group differences in performance IQ (in both the Block Design and Matrix Reasoning subtests measured a year earlier at 8 years of age) suggesting visual–spatial problems in the MD group, whereas the RD group performed better and was not significantly different from the control group.

Table 2 shows the preschool classification of children with RD, MD, and RD&MD. In this table, the columns refer to groups identified at 3½ years of age as being at familial risk for dyslexia or as having preschool language difficulties and low-risk (TD) controls. Data from a group of 15 children who were referred with concerns about language development, but who did not reach diagnostic criteria for language impairment at 3½ years of age, are included in the preschool group with language difficulties. Table 2 shows that, in children at familial risk for dyslexia, there was an elevated risk of RD relative to controls (29%; *χ*^2^ = 7.45, *p* = .01) but not of MD relative to controls (21%; *χ*^2^ = 3.40, *p* = .07). The risk for children who had preschool language difficulties (with or without familial risk of dyslexia) was significantly elevated relative to controls for both RD (34%; *χ*^2^ = 8.83, *p* = .003) and MD (39%; *χ*^2^ = 15.88, *p* = .001). The overlap with DLD, diagnosed at 8 years of age, is also striking. Although 20 of 61 children did not reach diagnostic criteria for either RD or MD, 6 children (9.8%) had RD, 12 children (19.7%) had MD, and (in line with our hypothesis) the children with concurrent DLD were the most likely group to experience RD&MD (23 cases, 37.7%). In short, in this high-risk sample, one third of cases diagnosed with DLD at 8 years of age experienced three disorders: RD, MD, and DLD.

**Table 2:** Numbers (and percentages) of children with RD, MD, RD&MD, and normal outcome according to preschool risk group (TD control, family risk of dyslexia, or preschool language difficulties).

Outcome at 9 years of age	Preschool history		
	TD control	FR dyslexia	Language difficulties
No impairment in reading or math	58 (81.7%)	55 (67.1%)	37 (52.8%)
RD only^a^	6 (8.5%)	10 (12.2%)	6 (8.6%)
MD only^b^	5 (7%)	3 (4.9%)	10 (14.3%)
RD&MD^c^	2 (2.8%)	14 (17.1%)	17 (24.2%)
Total	71	82	70

*Note.* TD, typically developing; FR dyslexia, familial risk of dyslexia; RD, reading disorder; MD, mathematics disorder; RD&MD, comorbid RD and MD.

a A total of 6 cases had developmental language disorder.

b A total of 12 cases had developmental language disorder.

c A total of 23 cases had developmental language disorder.

**Fig. 1:**
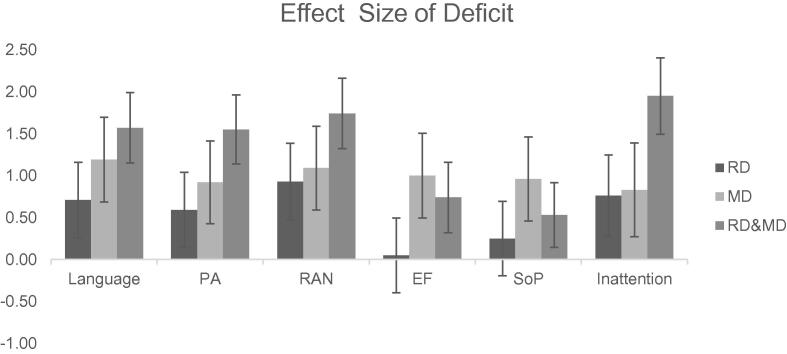
Effect size of deficit for reading disorder (RD), mathematics disorder (MD), and comorbid RD and MD (RD&MD) groups relative to controls (Cohen’s *d*) with 95% confidence intervals for language, phoneme awareness (PA), rapid automatized naming (RAN) digits, executive function (EF), speed of nonverbal processing (SoP), and ratings of inattention.

Table 3 shows the performance of the children with RD, MD, and RD&MD on factor scores derived from tests tapping different cognitive domains compared with the group of children in the sample whose reading and math skills were within the normal range (*F* values for between-group analyses of variance [ANOVAs] are provided in the middle column). The columns to the right show the data expressed in terms of the effect size of the deficits for each clinical group relative to the group with typical outcome (Cohen’s *d*). Given the relatively small sample size, it is important to focus on these effect sizes (see also Fig. 1).

**Table 3:** Means (and standard deviations) of cognitive measures and ratings of inattention (left columns) and effect sizes for deficits (Cohen’s *d*) (right columns).

	TD outcome (*n* = 150)	RD (*n* = 22)	MD (*n* = 18)	RD&MD (*n* = 33)	*F*(3, 218)	RD deficit [confidence interval]	MD deficit [confidence interval]	RD&MD deficit [confidence interval]
Language	.39 (.81)	−.18 (.73)_1_	−.56 (.67)_1,2_	−.90_2_ (.88)	27.83***	.71 [.26, 1.16]	1.19 [.69, 1.70]	1.57 [1.16, 2.00]
Phoneme awareness	.32 (.87)	−.19 (.78)_1_	−.49 (.96)_1_	−1.02 (.83)	24.39***	.59 [.14, 1.04]	.92 [.42, 1.41]	1.55 [1.14, 1.96]
								
RAN digits	.38 (.87)	−.41 (.68)_1_	−.57 (.81)_1,2_	−1.11 (.76)_2_	33.45***	.93 [.47, 1.38]	1.09 [.59, 1.59]	1.74 [1.31, 2.15]
Executive function	.20 (.88)_1_	.16 (.83)_1_	−.69 (1.00)_2_	−.48 (1.09)_2_	8.98***	.05 [−.40, .49]	1.00 [.49, 1.50]	.74 [.35, 1.12]
Speed of processing^a^	-.14 (.89)_1_	−.35 (.58)_1_	.73 (1.09)_2_	.34 (1.06)_2_	7.67***	.25 [−.20, .69]	−.96 [−1.46, .46]	−.53 [−.91, −.14]
Inattention^b^	.34 (.74)	−.23 (.90)_1_	−.27 (.72)_1_	−1.15 (.89)	31.40***	.76 [.28,1.25]	.83 [.27, 1.39]	1.95 [1.49, 2.40]

*Note.* All measures are factor scores except rapid automatized naming (RAN) digits and speed of processing. RD, reading disorder; MD, mathematics disorder, RD&MD, comorbid RD and MD. Means sharing the same subscript do not differ.

a Simple reaction time [RT(*z*)].

b Parent and teacher ratings on Strengths and Weaknesses of ADHD (attention-deficit/hyperactivity disorder) Symptoms and Normal Behavior Questionnaire (SWAN) (missing data imputed) (TD = 140, RD = 19, MD = 14, RD&MD = 29).

*** *p* < .001.

There is a stepwise pattern across groups for language, phoneme awareness, RAN, and ratings of inattention: TD control > RD > MD > RD&MD. All clinical groups were significantly impaired relative to controls on these measures. The pattern of performance was different for executive function and speed of processing: TD control > RD > RD&MD > MD. The RD and MD groups did not differ significantly in language, phoneme awareness, RAN digits, or ratings of inattention, but the RD group showed better performance on tests of executive skills and speed of processing. The pattern of performance can be seen more clearly by examining the effect sizes of the deficits relative to the control group (see Fig. 1); the effect sizes for the deficits in language, phoneme awareness, and RAN digits are large for the RD group, and the deficits in executive function and speed of processing are small to negligible. In contrast, the MD group showed large deficits across all tasks (including Block Design; see Table 1). The comorbid group was more impaired than either the RD or MD group in phoneme awareness, but the RD&MD group did not differ significantly from the MD group in language, RAN, executive function, or speed of processing. It is noteworthy that the deficit of this group in executive function and speed of processing was smaller than that of the MD-only group. All groups were rated as having attentional problems, and the comorbid group was the worst affected.

## Discussion

This study investigated reading and mathematics outcomes at 9 years of age for children recruited at 3½ years of age for being at high risk for reading and language difficulties. A novel aspect of the study is that it recruited children prior to formal instruction in reading and mathematics, allowing a window on early risk factors for specific learning disorders and their comorbidities, including familial risk. Moreover, in contrast to the majority of familial risk studies, a group of children with preschool language difficulties was included as well as a low-risk control group of children the same age, allowing particular consideration of the impact of poor language on learning number skills as well as on reading development.

Our focus was on the cognitive risk factors at 6 years of age associated with RD and MD identified 3 years later at 9 years of age. One specific aim was to identify shared risk factors that might account for the comorbidity between RD and MD. In line with our hypothesis, we found an elevated risk of both RD and MD in children at familial risk for dyslexia. These children were about three times more likely to develop RD, and twice as likely to develop MD, as controls. The risk was greater for children who had preschool language difficulties (with or without familial risk of dyslexia), for whom the risk of RD was three times higher, and the risk of MD was about four times higher, than in controls.

In this sample, 60% of children with RD had MD and 65% of children with MD had RD; however, it is important to acknowledge that the levels of comorbidity reported here are higher than those reported from population studies (e.g., Landerl & Moll, 2010) because it was a high-risk sample. Notwithstanding this, an important finding was the high rates of both RD and MD in children with oral language problems. Furthermore, the overlap with concurrent DLD, identified at 8 years of age, is striking, with 27% of the RD group, 67% of the MD group, and 70% of the RD&MD group having co-occurring DLD. It is also notable that nearly a third of the sample experienced three disorders: DLD, RD, and MD. These findings highlight the importance of taking levels of language disability into account when investigating cognitive deficits in children with either RD or MD.

Our hypotheses concerning shared risk factors and specific risks factors for specific learning disorders were partly confirmed. As expected, both RD and MD were associated with deficits in verbal skills (here defined by a language factor), in verbal processing speed (on a measure of RAN), and in parental ratings of inattention; however, in contrast to our hypothesis that phoneme awareness would be a specific deficit in RD, both groups showed deficits on this measure. Consistent with our hypothesis, the group with MD was impaired in executive function and visual–spatial skills, whereas these skills were in the normal range for the RD group; those with MD also had lower scores in speed of nonverbal processing. The comorbid group performed worse than the single-disorder groups on all measures except speed of nonverbal processing and executive function, for which the effect size of the deficit was smaller than that for the MD group. There was no evidence, based on a complementary series of 2 × 2 between-group analyses, of any interaction between RD and MD. Thus, cognitive risks were additive in the etiology of the comorbid group, in line with previous research analyzing the interaction between RD and MD (e.g., Cirino et al., 2015, Landerl et al., 2009, Moll et al., 2014, Raddatz et al., 2017, van der Sluis et al., 2004).

The findings of this at-risk study underline the fact that specific learning disorders, such as RD and MD, are the result of multiple risk factors shared across different behavioral disorders (Pennington, 2006). They align with the common liabilities model in confirming that poor language is a significant risk factor for RD and MD and their comorbidity. Furthermore, the comorbidity of each of the specific learning disorders (RD and MD) with DLD was extremely high, in line with reports of shared genetic risk factors operating for all three disorders (Haworth & Plomin, 2010). However, the current data set is small, precluding analysis of which of the risk measures have direct effects on developmental outcomes, and the causal significance of the findings remain equivocal.

The finding that RD is strongly associated with DLD is consistent with previous research (Bishop and Snowling, 2004, Catts et al., 2005); however, the finding that DLD was more strongly associated with MD was unexpected. Although this is a finding of considerable clinical importance, it needs to be borne in mind that the language deficits of the MD group were larger than those for the RD group, and this confounds interpretation.

In summary, the current findings suggest that the comorbidity between RD and MD arises because of shared risk factors, with language deficits being an important domain-general risk factor. In this study, there were also deficits in phoneme awareness and RAN common to both disorders, but whether these deficits comprise part of a unitary language factor cannot be determined. Arguably, these are pointers to dysfunction in language-related left hemisphere circuitry underpinning both reading and arithmetic (e.g., Peters and De Smedt, 2017, Prado et al., 2014). The additional deficits in executive skills, visual–spatial skills, and nonverbal processing point to broader impairments in MD, as predicted by reports of the involvement of bilateral parietal brain regions during processing numerosities (e.g., Dehaene, Piazza, Pinel, & Cohen, 2003). Finally, consistent with Willcutt et al. (2013), parents of children with reading and mathematics difficulties rated their children as inattentive, and ratings were highest for those with comorbid disorders.

The children recruited to the current sample were at high risk for reading and language disorders, and it follows that caution needs to be exercised before generalizing these findings to the general population. However, two findings are of particular importance: the high risk of MD in children who have experienced language difficulties (Cross et al., 2019) and the high risk of RD&MD in children with concurrent DLD. We conclude that assessments for either disorder should routinely consider the possible role of language and attentional difficulties and that interventions should take account of such problems. More specifically, children with preschool language difficulties should be monitored for emergent problems in reading and mathematics, and programs to strengthen their foundation for learning should be put in place (Hulme, Snowling, West, Lervåg, & Melby-Lervåg, 2020).

## References

[b0005] American Psychiatric Association (2013). Diagnostic and statistical manual of mental disorders (DSM-5).

[b0010] Bishop D.V.M. (2003). Test for Reception of Grammar (TROG-2).

[b0015] Bishop D.V.M., Snowling M.J. (2004). Developmental dyslexia and specific language impairment: Same or different?. Psychological Bulletin.

[b0020] Bishop D.V.M., Snowling M.J., Thompson P.A., Greenhalgh T., CATALISE-2 Consortium (2017). Phase 2 of CATALISE: A multinational and multidisciplinary Delphi consensus study of problems with language development: Terminology. Journal of Child Psychology and Psychiatry.

[b0025] Branum-Martin L., Fletcher J.M., Stuebing K.K. (2013). Classification and identification of reading and math disabilities: The special case of comorbidity. Journal of Learning Disabilities.

[b0030] Breckenridge K. (2008). August). Attention and executive function in Williams syndrome and Down’s syndrome [Apples task]. Paper presented at the Development of Executive Functions Workshop.

[b0035] Brownell R. (2000). Expressive and Receptive One-Word Picture Vocabulary Tests: Manual.

[b0040] Caron C., Rutter M. (1991). Comorbidity in child psychopathology: Concepts, issues and research strategies. Journal of Child Psychology and Psychiatry.

[b0045] Catts H.W., Adlof S.M., Hogan T.P., Weismer S.E. (2005). Are specific language impairment and dyslexia distinct disorders?. Journal of Speech, Language, and Hearing Research.

[b0050] Cirino P.T., Fuchs L.S., Elias J.T., Powell S.R., Schumacher R.F. (2015). Cognitive and mathematical profiles for different forms of learning difficulties. Journal of Learning Disabilities.

[b0055] Cross A.M., Joanisse M.F., Archibald L.M.D. (2019). Mathematical abilities in children with developmental language disorder. Language, Speech, and Hearing Services in Schools.

[b0060] Dehaene S., Piazza M., Pinel P., Cohen L. (2003). Three parietal circuits for number processing. Cognitive Neuropsychology.

[b0075] Durkin K., Mok P.L., Conti-Ramsden G. (2013). Severity of specific language impairment predicts delayed development in number skills. Frontiers in Psychology.

[b0080] Forum for Research Into Language and Literacy (2012). Diagnostic Test of Word Reading Processes.

[b0085] Göbel S.M., Snowling M.J. (2010). Number processing skills in adults with dyslexia. Quarterly Journal of Experimental Psychology.

[b0090] Haberstroh S., Schulte-Körne G. (2019). Clinical practice guideline: The diagnosis and treatment of dyscalculia. Deutsches Ärzteblatt international.

[b0095] Haworth C.M.A., Plomin R. (2010). Quantitative genetics in the era of molecular genetics: Learning abilities and disabilities as an example. Journal of the American Academy of Child & Adolescent Psychiatry.

[b0100] Hulme C., Nash H.M., Gooch D., Lervag A., Snowling M.J. (2015). The foundations of literacy development in children at familial risk of dyslexia. Psychological Science.

[b0105] Hulme C., Snowling M.J., West G., Lervåg A., Melby-Lervåg M. (2020). Children’s language skills can be improved: Lessons from psychological science for educational policy. Current Directions in Psychological Science.

[b0110] Hulme C., Stothard S.E., Clarke P., Bowyer-Crane C., Harrington A., Truelove E., Snowling M.J. (2009). York Assessment of Reading for Comprehension: Early Reading.

[b0115] Joyner R., Wagner R. (2019). Co-occurrence of reading disabilities and math disabilities: A meta-analysis. Scientific Studies of Reading.

[b0120] Koponen T., Aro M., Poikkeus A.M., Niemi P., Lerkkanen M.K., Ahonen T., Nurmi J.E. (2018). Comorbid fluency difficulties in reading and math: Longitudinal stability across early grades. Exceptional Children.

[b0125] Koponen T., Eklund K., Heikkilä R., Salminen J., Fuchs L., Fuchs D., Aro M. (2020). Cognitive correlates of the covariance in reading and arithmetic fluency: Importance of serial retrieval fluency. Child Development.

[b0130] Korpipää H., Koponen T., Aro M., Tolvanen A., Aunola K., Poikkeus A.-M., Nurmi J.-E. (2017). Covariation between reading and arithmetic skills from Grade 1 to Grade 7. Contemporary Educational Psychology.

[b0135] Landerl K., Bevan A., Butterworth B. (2004). Developmental dyscalculia and basic numerical capacities: A study of 8-9-year-old students. Cognition.

[b0140] Landerl K., Fussenegger B., Moll K., Willburger E. (2009). Dyslexia and dyscalculia: Two learning disorders with different cognitive profiles. Journal of Experimental Child Psychology.

[b0145] Landerl K., Moll K. (2010). Comorbidity of learning disorders: Prevalence and familial transmission. Journal of Child Psychology and Psychiatry.

[b0150] LeFevre J.-A., Fast L., Skwarchuk S.-L., Smith-Chant B.L., Bisanz J., Kamawar D., Penner-Wilger M. (2010). Pathways to mathematics: Longitudinal predictors of performance. Child Development.

[b0160] Moll K., Kunze S., Neuhoff N., Bruder J., Schulte-Körne G. (2014). Specific learning disorder: Prevalence and gender differences. PLoS One.

[b0170] Moll K., Snowling M.J., Gobel S.M., Hulme C. (2015). Early language and executive skills predict variations in number and arithmetic skills in children at family-risk of dyslexia and typically developing controls. Learning and Instruction.

[b0175] Moll K., Snowling M.J., Hulme C. (2020). Introduction to the special issue “Comorbidities Between Reading Disorders and Other Developmental Disorders”. Scientific Studies of Reading.

[b0180] Nash H.M., Hulme C., Gooch D., Snowling M.J. (2013). Preschool language profiles of children at family risk of dyslexia: Continuities with SLI. Journal of Child Psychology and Psychiatry.

[b0185] Peng P., Wang C., Namkung J. (2018). Understanding the cognition related to mathematics difficulties: A meta-analysis on the cognitive deficit profiles and the bottleneck theory. Review of Educational Research.

[b0190] Pennington B.F. (2006). From single to multiple deficit models of developmental disorders. Cognition.

[b0195] Peters L., De Smedt B. (2017). Arithmetic in the developing brain: A review of brain imaging studies. Developmental Cognitive Neuroscience.

[b0200] Peterson R.L., Boada R., McGrath L.M., Willcutt E.G., Olson R.K., Pennington B.F. (2017). Cognitive prediction of reading, math, and attention: Shared and unique influences. Journal of Learning Disabilities.

[b0205] Pickering S., Gathercole S. (2001). Working Memory Test Battery for Children (WMTB-C).

[b0210] Prado J., Mutreja R., Booth J.R. (2014). Developmental dissociation in the neural responses to simple multiplication and subtraction problems. Developmental Science.

[b0215] Purpura D.J., Hume L., Sims D.M., Lonigan C.J. (2011). Early literacy and early numeracy: The value of including early literacy skills in the prediction of numeracy development. Journal of Experimental Child Psychology.

[b0220] Raddatz J., Kuhn J.T., Holling H., Moll K., Dobel C. (2017). Comorbidity of arithmetic and reading disorder: Basic number processing and calculation in children with learning impairments. Journal of Learning Disabilities.

[b0225] Semel E., Wiig E.H., Secord W. (2006). Clinical Evaluation of Language Fundamentals-Fourth Edition UK (CELF-4UK).

[b0230] Snowling M.J., Nash H.M., Gooch D.C., Hayiou-Thomas M.E., Hulme C., Wellcome, Language & Reading Project Team (2019). Developmental outcomes for children at high risk of dyslexia and children with developmental language disorder. Child Development.

[b0235] Swanson H.L., Jerman O. (2006). Math disabilities: A selective meta-analysis of the literature. Review of Educational Research.

[b0240] Swanson J.M., Schuck S., Mann Porter M., Carlson C., Hartman K., Sergeant J.A., Wigal T. (2012). Categorical and dimensional definitions and evaluations of symptoms of ADHD: History of the SNAP and the SWAN Rating Scales. International Journal of Educational and Psychological Assessment.

[b0245] Thapar A., Cooper M., Rutter M. (2017). Neurodevelopmental disorders. *Lancet*. Psychiatry.

[b0250] van Bergen E., de Jong P.F., Maassen B., van der Leij A. (2014). The effect of parents’ literacy skills and children’s preliteracy skills on the risk of dyslexia. Journal of Abnormal Child Psychology.

[b0255] van der Sluis S., de Jong P.F., van der Leij A. (2004). Inhibition and shifting in children with learning deficits in arithmetic and reading. Journal of Experimental Child Psychology.

[b0260] Wechsler D. (2003). Wechsler Intelligence Scale for Children (WISC).

[b0265] Wechsler D. (2005). Wechsler Individual Achievement Test 2nd UK Edition (WIAT II).

[b0270] Willcutt E.G., Petrill S.A., Wu S., Boada R., DeFries J.C., Olson R.K., Pennington B.F. (2013). Comorbidity between reading disability and math disability: Concurrent psychopathology, functional impairment, and neuropsychological functioning. Journal of Learning Disabilities.

